# Experimental Study on the Mechanical Properties of Perfobond Rib Shear Connectors with Steel Fiber High Strength Concrete

**DOI:** 10.3390/ma14123345

**Published:** 2021-06-17

**Authors:** Fangwen Wu, Shuo Liu, Chengfeng Xue, Kangkang Yang, Yanpeng Feng, Hao Zhang

**Affiliations:** 1 School of Highway, Chang’an University, Xi’an 710064, China; wufangwen@chd.edu.cn (F.W.); 2019221009@chd.edu.cn (S.L.); 2019221027@chd.edu.cn (Y.F.); 2 School of Civil Engineering, Xijing University, Xi’an 710123, China; chfxue2008@163.com; 3 School of Civil Engineering, Harbin Institute of Technology, Harbin 150090, China; kakaer@hit.edu.cn; 4 State Key Laboratory of Mechanical Behavior and System Safety of Traffic Engineering Structures, Shijiazhuang Tiedao University, Shijiazhuang 050043, China

**Keywords:** perfobond rib shear connector, steel fiber high strength concrete, push out test, load-slip curve, shear capacity equation

## Abstract

Perfobond rib (PBL) shear connectors, made up of the perforated steel plates with the penetrating rebars passing through the holes, are extensively adopted in steel-concrete composite structures for their excellent performance. The adequate understanding of mechanical properties for PBL connectors is of great significance for their reasonable design. In this study, a push out experiment, including 12 specimens with the parameters of concrete strength, diameter of penetrating rebars and the number of holes on perforated steel plate, was performed to explore the mechanical behavior of PBL connectors with steel fiber high strength concrete (SFHSC). The experimental results showed that the shear capacity of the PBL connectors increased with the increase in concrete strength, diameter of the penetrating rebars and the number of holes. Furthermore, a general prediction formula for the shear capacity of PBL connectors was developed, which considers the shear contribution of concrete dowels, concrete end-bearing, interfacial bonding between the perforated steel plates and concrete and the penetrating rebars as well as the enhancement effect of steel fibers. The prediction results of the equation are in good agreement with the experimental data and could provide a reference for the design of PBL connectors.

## 1. Introduction

The use of steel-concrete composite structures in civil engineering has seen rapid growth in the past decades due to their excellent mechanical properties, convenient construction performance and outstanding economic features [1]. In order to fully utilize the respective properties of steel and concrete, it is necessary to resist the slip between steel and concrete through shear connectors to ensure a reliable connection between the two materials [2]. As the most commonly used shear connectors for composite structures, headed studs have the advantages of good ductility, simple preparation and easy construction, but also have the disadvantages of insufficient fatigue performance and harsh requirements for installation equipment [3,4]. In contrast to the stud connectors, perfobond rib shear connectors, proposed in 1987s by Leonhardt [5] and also known as PBL shear connectors, are widely adopted for their convenient construction, excellent bearing capacity and superior anti-fatigue performance [6,7].

To date, the exploration for the mechanical behavior of PBL connectors with conventional concrete have been executed based on push out tests. Oguejiofor and Hosain [8] reported that the interlocking effect of adjacent holes of perforated plates could be eliminated by a spacing greater than 2.25 times the hole diameter, and further developed a prediction formula for the shear capacity of PBL connectors. With the omission of the end concrete, He et al. [9] adopted the plug-in specimens to explore the shear transfer mechanism of PBL connectors, and proposed a formula for the prediction of the shear resistance of PBL connectors. Zheng et al. [10] discovered that the load-slip curve of PBL connectors was significantly influenced by the hole geometry, the concrete strength and the configuration of penetrating rebars and presented a prediction formula for the load-slip curve. Zhan et al. [11] found that external pressure could improve the shear stiffness and strength of PBL connectors, but the improvement was not significant.

In recent years, various problems have been identified in the service of PBL connectors with conventional concrete. Specifically, it is difficult to ensure the casting quality of coarse aggregate concrete in the joint area during the construction process. In addition, the shrinkage of conventional concrete can result in the segregation between the steel plate and concrete [12]. These problems would cause degradation of the mechanical performance of PBL connectors. Ultra high-performance concrete (UHPC) has become a solution to these problems due to its ultra-high strength, better ductility and less shrinkage [13], and the mechanical performance of the PBL connectors with UHPC have attracted much scholarly attention. Kang et al. [14] demonstrated that the increase in concrete strength and the hole number could enhance the shear strength of connectors. He et al. [15] revealed that UHPC can significantly increase the shear capacity of PBL connectors and retard the cracking of concrete, and established a prediction formula for the shear strength of connector. He et al. [16] designed shear connectors wrapped by steel chambers filled with UHPC, and pointed out that the confinement of UHPC could improve the shear performance of PBL connectors. Duan et al. [17] observed that during the failure of the PBL connectors with UHPC, there were few cracks in the specimens and the overall stiffness was maintained at a high level, which is different from those with conventional concrete.

With the application of UHPC in PBL connectors, some problems have been gradually discovered. The construction process of UHPC materials is complicated, which is not conducive to large-scale preparation at construction sites [18]. In addition, the small water-to-binder ratio of UHPC causes large autogenous shrinkage deformation, jeopardizing the normal use of the structure [19]. Moreover, the large demand for cement of UHPC results in expensive production costs. Some scholars have discovered that the utilization of steel fiber high strength concrete (SFHSC) with low strength (around 80 MPa) in steel-concrete joints could effectively compensate for the defects of convention concrete and UHPC [20,21]. SFHSC with the strength between conventional concrete and UHPC has a more convenient construction process and less autogenous shrinkage deformation than UHPC and superior mechanical and durability properties than conventional concrete [22,23]. Moreover, the reliable bond between steel fibers and concrete can inhibit the cracking and damage of concrete structures and enhance the elastic deformation capacity of concrete, thus greatly improving the shear resistance as well as the ductility of concrete structures [24].

Even though numerous studies have been conducted on PBL connectors, there are still many problems to be solved. The experimental studies on the mechanical properties of PBL connectors are mostly focused on the specimens adopting conventional concrete or UHPC [25,26], while there are few experimental investigations on PBL connectors with SFHSC with low strength, resulting in a lack of understanding for the performance of such connectors. Moreover, most of the existing formulas for calculating the shear capacity of PBL connectors do not adequately consider the contributions of concrete dowels, penetrating rebars, concrete end-bearing and interface bond between the perforated steel plates and concrete [27,28]. For those few formulas that do consider the shear contributions of the four components, they were obtained from regression analysis based on experimental data of PBL connectors with conventional concrete [29] and cannot account for the shear capacity enhancement effect of steel fibers. Therefore, they cannot be applied for the prediction of the shear capacity of PBL connectors with SFHSC either.

To investigate the mechanical properties of PBL connectors with SFHSC, 12 push out specimens, with the variable parameters of concrete strength, diameter of the penetrating rebars and number of holes, were designed and tested. At first, the failure modes and the characteristics of the load-slip curves of the PBL connectors with SFHSC were analyzed. Afterwards, the influence of each specimen parameter on the mechanical properties of the connectors, include shear capacity, ductility and shear stiffness, was discussed based on the test results. Finally, a general equation for calculating the shear capacity of PBL connectors was established. The proposed equation fully considers the shear contributions of concrete dowels, concrete end-bearing, interface bond between perforated steel plates and concrete and penetrating rebars, as well as the reinforcing effect of the steel fibers. Furthermore, the validation for the accuracy and applicability of the equation was performed.

## 2. Experimental Program

### 2.1. Specimen Design

**Figure 1:**
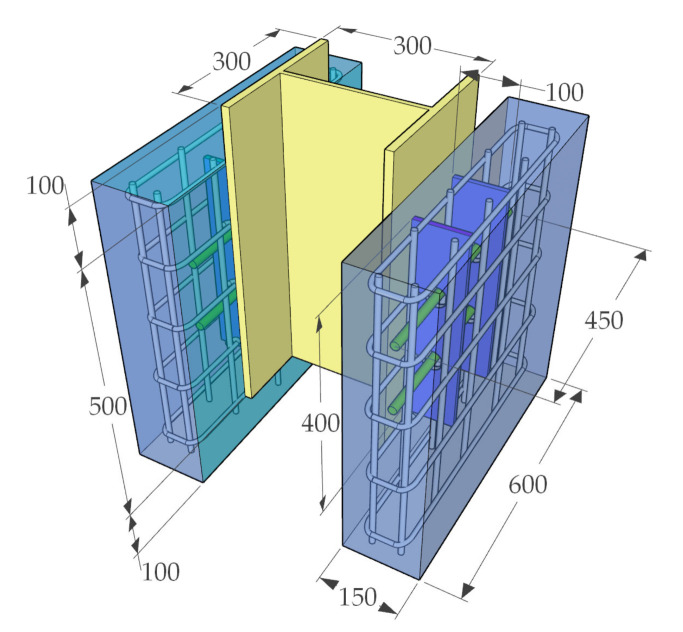
Dimensions and details of specimens (unit: mm).

**Figure 2:**
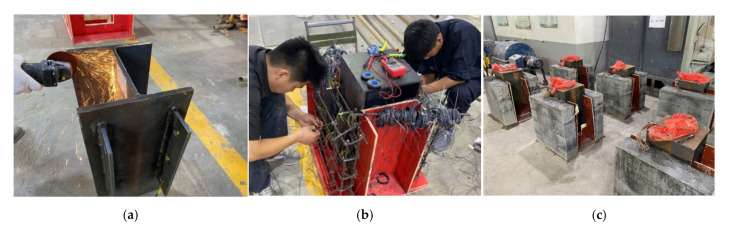
Fabrication of test specimens: (**a**) PBL processing; (**b**) rebar tying; (**c**) completed casting.

In order to investigate the mechanical properties of PBL connectors with SFHSC in depth, the push out test including 12 specimens was conducted and the variable parameters consist of concrete strength, diameter of penetrating rebars and the number of holes on perforated steel plates. The specimens adopted in this study were designed on the basis of Eurocode 4 [30] as depicted in Figure 1 and fabricated with reference to Standard DB 41/T 696-2011 [31] as shown in Figure 2. Specifically, each specimen was made up of a 600 mm long structural steel with the section of HW300 mm × 300 mm × 10 mm × 15 mm and a 600 mm × 600 mm × 150 mm concrete slab on both flanges of the steel section. The perforated steel plates with the height, width, thickness and hole diameter of 400 mm, 100 mm, 10 mm and 40 mm, respectively, were embedded in concrete slab, and the center distance of the steel plates on the same side was 200 mm. The 450 mm length of penetrating rebars were placed in each hole. The diameter of stirrups and hoisting bars were 8 mm and 10 mm, respectively. Furthermore, in order to ensure the welding quality of the specimens, the perforated steel plates and H-beam were welded together with J506 electrode. The C50, C80 and C100 concrete were adopted in this test, and the steel fibers with the volume content, length and diameter of 2%, 13 mm and 0.2 mm, respectively, are present in C80 and C100 concrete. The number of holes on the perforated steel plates ranged from 1 to 3. The diameter of penetrating rebars (*d*) includes 12, 16 and 20 mm. The specific labels and parameters of each specimen were summarized in Table 1.

For all specimens, the Q345D structural steel with a thickness of 10 mm was adopted for the perforated steel plates and H-beams, and had the nominal yield strength, ultimate strength and elastic modulus of 345 MPa, 470 MPa and 209 GPa, respectively. The specimens were reinforced with HRB400 steel bars whose yield strength, ultimate strength and elastic modulus were 400 MPa, 570 MPa and 200 GPa, respectively. At the same time of each specimen was fabricated, three concrete cubic blocks with specifications of 150 mm × 150 mm × 150 mm were poured and cured. The real cubic strength (*f*_cu_) and elastic modulus (*E*_c_) of concrete was determined from concrete cubic tests after the standard 28 day setting as displayed in Table 1.

### 2.2. Loading Setup and Measuring System

**Figure 3:**
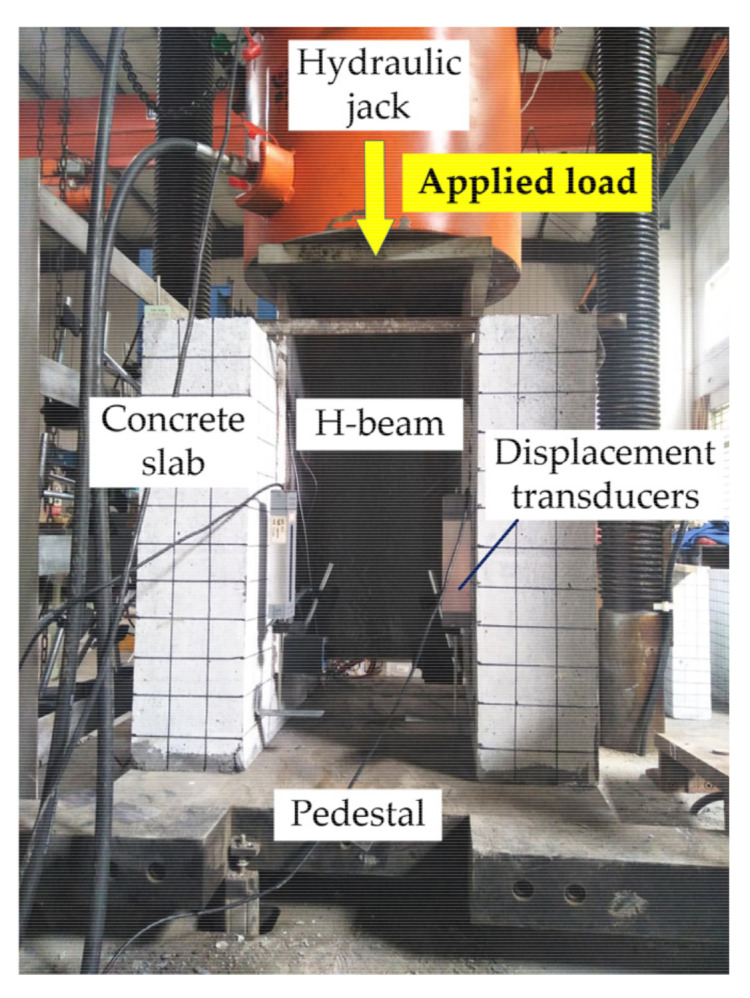
Loading setup.

The loading setup is shown in Figure 3. Specifically, the microcomputer-controlled compression-shear fatigue test loading system produced by Tianshui Hongshan testing Machine Co., Ltd. (Tianshui, Gansu Province, China) with a specific model of PLU-1000 was adopted, and the load was applied to the top of the H-beam via the hydraulic jack. Before the loading test, the specimens were painted and the grid of 50 mm × 50 mm was drawn to observe the crack development during the test. In order to ensure the uniform load distribution, the top of the H-beam was polished and a 300 mm × 300 mm steel plate was welded between the H-beam and hydraulic jack. In addition, the specimen was placed on the pedestal and the fine sand was evenly sprinkled between the pedestal and the concrete slabs. Four linear variable displacement transducers (LVDTs) were equipped on each specimen to monitor the relative displacement between concrete slab and H-beam during the test. The LVDTs were symmetrically arranged front-to-back on four faces of the concrete slab, and four angle plates were glued onto the H-beam below the LVDTs’ pointer. 

The loading process includes preloading stage and formal loading stage. The elastic bearing capacity of the shear connectors was calculated in the light of the finite element analysis and design theory, and the 50 kN was loaded as a preload to eliminate the influence of gaps and various unstable factors in each part of the loading system. After 10-min preloading, the readings of each measuring point were recorded as the initial state of the test. In the formal loading stage, the specimens were loaded by stages through the failure bearing capacity of the existing formula and the 20 kN was adopted as the load of each stage. A load increasing rate of 5 kN/min was adopted in this test. Whenever the load in each stage reaches the predetermined value, the load was kept constant for 5 min to make the deformation sufficient. In addition to the automatic data acquisition device, manual recording was also adopted in this test to ensure the collection of measurement data under all levels of load. The test ended when the loading device automatically returns oil and unloads.

## 3. Analysis of Test Results

### 3.1. Damage and Failure Modes

**Figure 4:**
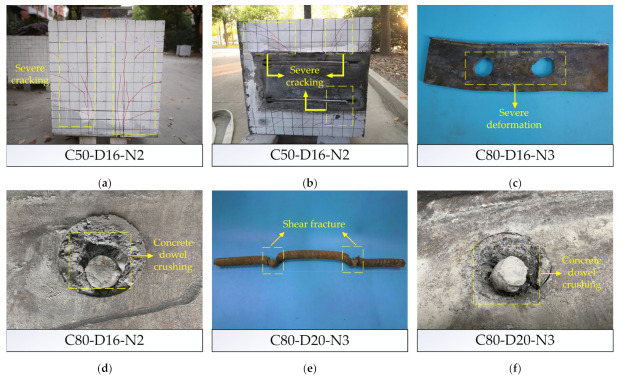
Typical failure modes of PBL connectors: (**a**) outside of concrete slab; (**b**) inside of concrete slab; (**c**) perforated steel plate; (**d**) concrete dowel; (**e**) penetrating rebar; (**f**) concrete dowel.

According to the observation for the failure appearance of each specimen, the failure modes of PBL connectors designed in this test could be divided into four types: severe cracking of concrete slabs, yield of perforated steel plates, crushing of concrete dowels and fracture of penetrating rebars. Each PBL connector failure correspond to one or more failure modes. The four types of failure modes could be clearly characterized based on the analysis of the damage morphology of the four specimens in Figure 4: (1) Specimen C50-D16-N2 has serious concrete slab cracking as shown in Figure 4a,b. The reason for this failure can be explained by that the concrete around the perforated steel plates bear the shear force and the reaction force exerted by the steel plates, resulting in the complex force of concrete in this area. The concrete strength of this specimen is relatively low, thus the cracks appeared in the plane parallel to the perforated steel plates and swelled rapidly, resulting in the destruction of peripheral concrete. (2) Figure 4c shows the severe deformation of the perforated steel plates in Specimen C80-D16-N3, while the concrete dowels and penetrating rebars failed to give full play to their roles since there was a slight cracking and deformation but not damage in them. (3) Specimen C80-D16-N2 experienced crushing failure of the concrete dowels, as shown in Figure 4d, while there is no obvious deformation and damage in the penetrating rebars and perforated steel plates. Concrete dowel is the main bearing member of PBL connectors, and there is a mutual force transfer between that with both the steel plates and the penetrating rebars, so its real stress state is more complex. Under the action of the large force, the concrete dowels were damaged. (4) The failure morphology of Specimen C80-D20-N3 is shown in Figure 4e,f. Shear failure occurs in the penetrating rebars, and the concrete dowels are also accompanied by a certain failure imagination, the two are in a state of coexistence, which is because after the destruction of concrete dowels, most of the loads they carried were transferred to the penetrating rebars and the excessive shear force led to the destruction of the rebars.

It is worth pointing out that for the specimens with SFHSC, the failure modes are basically not severe cracking of concrete slab. This phenomenon can be explained in two ways, on the one hand, high strength concrete could provide excellent ductility, on the other hand, the adoption of longer penetrating rebars significantly reduces the stresses in the concrete. Furthermore, the cracks of the specimens using SFHSC are mainly concentrated in the middle and bottom parts of the side of concrete slab. Therefore, when the PBL connectors with SFHSC are adopted in practical engineering, these parts can be strengthened in advance, which can effectively prolong the service life of the connectors.

### 3.2. Load-Slip Curves

**Figure 5:**
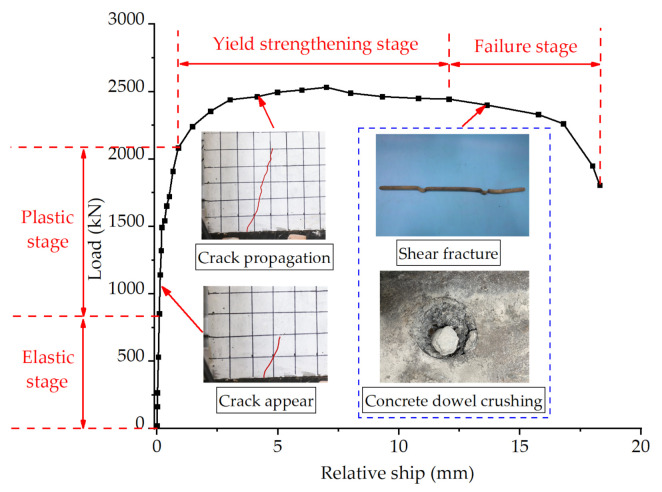
Typical load-slip curve from Specimen C80-D12-N3.

The relative slip between the H-beam and concrete slab was generated as the load increased, which was collected by four LVDTs in this test. Taking the load-slip curve of Specimen C80-D12-N3 displayed in Figure 5 as an example, the failure process of the PBL connector could be basically divided into four stages: (1) **Elastic stage**. At the initial stage of loading, the PBL shear connectors displayed significant stiffness with virtually no relative slip between the H-beam and concrete slab generation. The stress in the specimen was quite small and the load increased linearly and rapidly. Meanwhile, there was mutual compaction between the concrete dowels and accompanied by a sizzling sound. (2) **Plastic stage**. The stress in the specimen gradually increased with the increase of load. The cracks in concrete gradually developed, and the stiffness of the connectors decreased, resulting in a reduction in the slope of load-slip curve. At this time, the outer surface of concrete and the junction of H-beam and concrete slab began to crack; (3) **Yield strengthening stage**. As the load increased, the growth rate of load-slip curve decreased further. Due to the high strength and good ductility of C80 concrete, cracks were almost no longer developed. There was a degree of deformation in the penetrating rebars and perforated steel plates under the action of shear force, and the stiffness of the connector was correspondingly reduced. Furthermore, this stage lasted for a long time due to the good ductility of the penetrating rebars and perforated steel plates, as well as the bonding force between steel fibers and concrete. (4) **Failure stage**. When the load reached the ultimate value, the PBL connector failed and the load-slip curve decreased rapidly. Corresponding to Specimen C80-D12-N3, the failure mode is the shear fracture of penetrating rebars accompanied by the crushing of concrete dowels.

**Figure 6:**
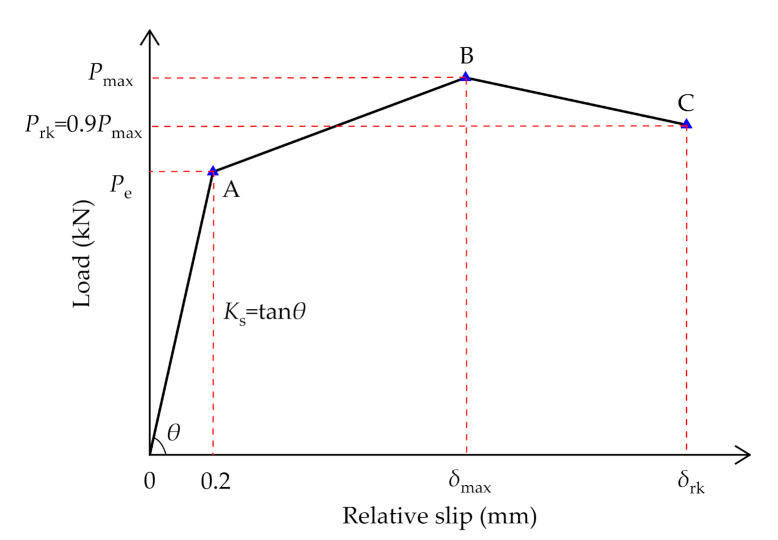
Definition of characteristic value for Load-slip curve.

The test results of all the specimens in this test were summarized with reference to Eurocode 4 [30] as presented in Table 2. Specifically, *P*_max_ and *P*_rk_ denote the maximum load and characteristic load of ductility (*P*_rk_ = 0.9 *P*_max_), respectively, and *δ*_max_ and *δ*_rk_ denote the corresponding relative slip, respectively, in which *δ*_rk_ was the index to reflect the ductility of connectors. In addition, *K*_s_ denotes the shear stiffness of the PBL connectors in the initial loading stage. As displayed in Figure 6, *K*_s_ is specified as the slope of the connection between the origin and point A that corresponds to a relative slip of 0.2 mm, and points B and C were determined by *P*_max_ and *P*_rk_, respectively.

### 3.3. Results Analysis

#### 3.3.1. Influence of Concrete Strength

**Figure 7:**
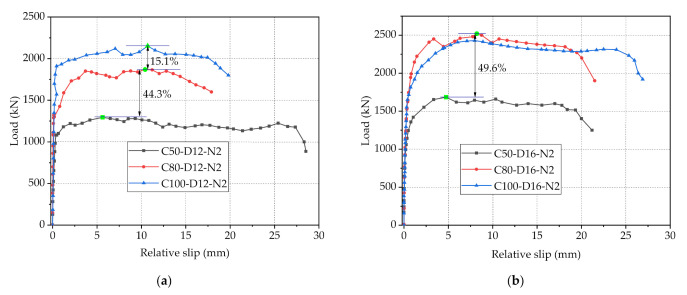
Load-slip curves of specimens with different concrete strength: (**a**) 12-mm-diameter rebar and 2-hole steel plate; (**b**) 16-mm-diameter rebar and 2-hole steel plate.

The load-slip curves of Specimens C50-D12-N2, C80-D12-N2 and C100-D12-N2 are shown in Figure 7a, it can be seen that concrete strength has a significant effect on the shear performance of PBL connectors. Specifically, as the concrete strength increased from 50 MPa to 80 MPa and continues to 100 MPa, the maximum loads of the connectors varied from 1295.3 to 1868.9 kN and continues to 2150.9 kN, increasing by 44.3% and 15.1%, respectively, and the initial shear stiffness increased by 69.2% and 30.6%, respectively. This is mainly because the concrete dowel is one of the main contribution parts for the shear strength of connectors, the improvement of concrete strength can effectively improve the shear capacity of concrete dowels and further improve the maximum bearing capacity of PBL connectors. However, with the increase of concrete strength, the growth magnitude of shear capacity of connectors becomes smaller and smaller. This may be due to the high concrete strength resulting in a strong shear capacity of the concrete dowels, in this case the shear capacity of the connectors is mainly determined by other parameters. Consequently, the influence magnitude of concrete strength on the bearing capacity of the connectors decreases gradually. Furthermore, for C80 and C100 concrete, the steel fibers with uniform distribution would affect the shear performance of the connectors to some extent. To be specific, the bonding force between steel fibers and high strength concrete can effectively improve the ductility of concrete and restrain the expansion of existing cracks and the formation of new cracks. Moreover, the steel fibers in the concrete dowels can provide strong binding force for the concrete and delay the damage of the concrete dowels [32]. As a result, the specimens have a prolonged plastic phase.

A noteworthy phenomenon observed in this test as shown in Figure 7b is that in terms of Specimens C50-D16-N2, C80-D16-N2 and C100-D16-N2, as the concrete strength increased from 50 MPa to 80 MPa, the anti-slip maximum load and initial shear stiffness increased by 49.6 and 23.3%, respectively, and the improvement effect was obvious. However, as the strength of concrete increased from 80 to 100 MPa, the maximum load varied quite slightly. This phenomenon may be due to the large diameter of the penetrating rebars results in the area of the concrete dowels is too small, leading to the premature destruction of the concrete dowels during the loading process. At this time, the increase of concrete strength has no great improvement on the shear capacity of PBL connectors. Therefore, when the rebar diameter is large and the concrete strength reaches a certain value, its continued increase has little effect on the shear capacity of connectors. In other words, merely increasing the strength of concrete does not necessarily improve the shear capacity of PBL connectors significantly and consistently.

#### 3.3.2. Influence of Diameter of Penetrating Rebar

**Figure 8:**
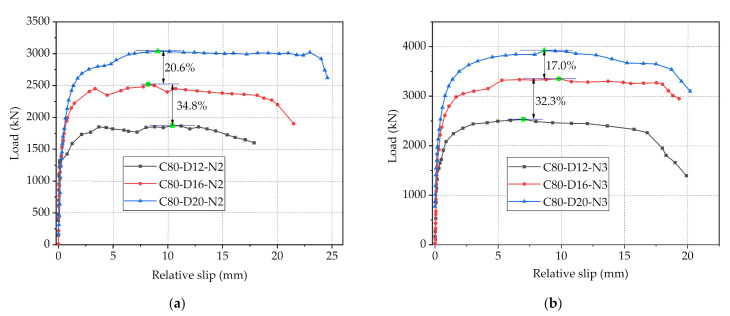
Load-slip curves of with different diameters of penetrating rebar: (**a**) C80 concrete and 2-hole steel plate; (**b**) C80 concrete and 3-hole steel plate.

The load-slip curves of specimens with C80 concrete and 2/3 holes on perforated steel plates, as depicted in Figure 8, were adopted to compare the variation brought by different diameters of penetrating rebars on the shear strength of PBL connectors. It can be found from Figure 8a that as the diameter of the penetrating rebars, respectively, increased from 12 mm to 16 mm and from 16 to 20 mm, the maximum loads of the connectors increased by 34.8 and 20.6%, respectively, while there is no significant variation in initial shear stiffness. This indicates that the increase in the diameter of the penetrating rebars would obviously improve the shear capacity of the connectors, which is mainly because that as the main contributor to the shear resistance of PBL connectors, the increase in diameter of the penetrating rebars can effectively improve their shear strength [33]. However, with the increase of the diameter of penetrating rebars, the increase magnitude of the maximum loads of connectors decreased gradually, which can be explained by that the decrease in the area of the concrete dowels due to the increase in the diameter of the penetrating rebars has a negative impact on the shear resistance of PBL connectors. Furthermore, in the case of a large diameter of the penetrating rebars, the area of the concrete dowels is tiny, at which point the concrete dowel shear capacity remains small despite the greater concrete strength. In general, in the design of PBL connectors, there should be a reasonable trade-off between the increase of penetrating rebar diameter and the reduction of concrete dowel area, in this way, the increase of rebar diameter can enhance the bearing capacity of connectors more obviously. Additionally, the laws reflected by the load-slip curves of the specimens with C80 concrete and 3 holes shown in Figure 8b are consistent with those in Figure 8a.

#### 3.3.3. Influence of Number of Holes

**Figure 9:**
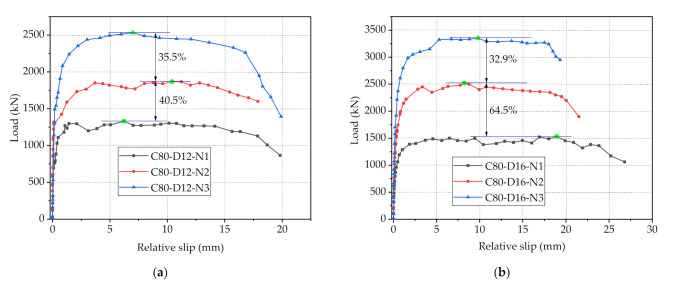
Load-slip curves of with different spacings of steel plate: (**a**) C80 concrete and 12-mm-diameter rebar; (**b**) C80 concrete and 16-mm-diameter rebar.

The load-slip curves of Specimens C80-D12-N1, C80-D12-N2 and C80-D12-N3 are shown in Figure 9a, it can be seen that with the increase of the number of holes from 1 to 2 and then to 3, the maximum loads of PBL connectors increased from 1330.3 to 1868.9 kN and then to 2532.2 kN, respectively, increasing by 40.5 and 35.5%, respectively. In addition, the initial stiffness of the specimens also grew as the number of holes increases. The improvement effect of the increase of hole number on the shear capacity and initial shear stiffness of PBL connectors is obvious, which can be explained by that the increase of the number of holes on steel plates leads to an increase in the concrete dowel area, causing a more uniform distribution of shear forces. This leads to an increase to the shear capacity and ductility of the connectors, which are reflected in the load-slip curve as an increasing of the curve height and a lengthening of the plastic phase, respectively. Nevertheless, the growth rate gradually decreased with the increase of the number of holes, and this is mainly owing to the increase of the number of holes would lead to the decrease of the cross-sectional area and volume of perforated steel plates, and then weaken the stiffness of steel plates, resulting to the decrease of the shear capacity of the connectors. The variation in the effect of increasing the number of holes on the shear resistance of the connectors can be more clearly observed from the load-slip curves of Specimens C80-D16-N1, C80-D16-N2 and C80-D16-N3, as shown in Figure 9b. With the number of holes varied from 1 to 2, the shear capacity of the connectors improved by 64.5%, which is noticeable. However, when the number of holes increased from 2 to 3, the shear capacity of the connectors increased by 32.9%, basically only half of 64.5%. Therefore, in the design of shear connectors, the number of holes is not the more the better, it is recommended to comprehensively consider other parameters of the connectors to design the number of holes, in order to maximize the area of the concrete dowels and minimize the impact on the stiffness of the steel plate stiffness.

## 4. Shear Capacity Equation for PBL Connectors

### 4.1. Existing Shear Capacity Equations for PBL Connectors

The reasonable prediction for the shear capacity of PBL connectors through theoretical calculation is a prerequisite for their rational design in practical engineering. Until now, various scholars have proposed formulas for predicting the shear capacity for PBL connectors. In this section, four representative formulas, respectively, proposed are summarized in Table 3.

It can be observed from the comparisons of prediction results (*V*_cu_) based on Equations (1)–(4) and the experimental data, as displayed in Table 4, that the predictions of these equations for the shear capacity of the PBL connectors in this test are all on the small side, with the ratio of prediction results to experimental ones ranging from 0.36 to 0.87. The reason for the prediction errors of Equations (1)–(3) may lie in the fact that they were derived based on the data obtained from the specimens adopting relatively low strength concrete without steel fibers mixed, thus they do not include the enhancement effect of steel fibers on the shear capacity. In terms of Equations (1), (3) and (4), these equations were estimated according to the test results of PBL connectors with a single steel plate on each side, while the PBL connectors in this study employ double steel plates. The working performance of two perforated plates distributed on the same side would have a certain degree of mutual influence and is not a simple superposition of shear resistance, which also leads to that the prediction results do not match well with the ones of this experiment to some extent. Additionally, another important reason is that the tests on which these formulas are based differ to a greater or lesser extent from the form of the specimens adopted in this test (e.g., there is no concrete end-bearing in the specimens from which Equation (4) was derived).

### 4.2. Composition of Shear Capacity for PBL Connectors

The prediction results of the existing representative formulas for PBL shear connectors do not agree well with the experimental data, mainly owing to that that the shear contribution of each component is not fully and accurately considered. Therefore, in order to accurately evaluate the shear capacity of PBL connectors with SFHSC, further analysis of the shear capacity of the connectors is imperative to fully and properly account for the contributions of each factor.

The shear capacity of PBL connectors is mainly contributed by concrete dowels, concrete end-bearing, interface bond between perforated steel plates and concrete and penetrating rebars, in which the effect of steel fibers could be reflected in the former three contributions. Thus, the formula for shear capacity could be generally expressed as:

(5) $$V_{u}=V_{\mathrm{cd}}+V_{\mathrm{eb}}+V_{\mathrm{ib}}+V_{\mathrm{pr}}$$

where *V*_cd_, *V*_eb_, *V*_ib_ and *V*_pr_ denote the shear capacity contributed by concrete dowels, concrete end-bearing, interface bond and penetrating rebars, respectively (N).

#### 4.2.1. Contribution of Concrete Dowels

When the PBL connector is subjected to shear force, the concrete dowels would be subjected to the pressure of the steel plates, penetrating rebars and adjacent concrete at the same time, causing a quite complex force state [36]. The contribution of the concrete dowels without steel fibers to the shear capacity of the PBL connector can be expressed as:

(6) $$V_{\mathrm{cd}}=2f_{\mathrm{cs}}A_{c}{}_{d}$$

where *f*_cs_ denotes the shear strength of the concrete under multiaxial stresses (MPa) and can be calculated by *f*_cs_ = 0.25*f*_c_, in which *f*_c_ denotes the prismatic strength of the concrete and can be obtained by *f*_c_ = 0.8*f*_cu_; and *A*_cd_ denotes the sectional area of the concrete dowel (mm^2^).

The shear strength of the concrete dowels in PBL connector is increased due to the action of external pressure [37], thus the contribution of that could be further expressed by introducing a strengthening coefficient *ψ*_1_ as:

(7) $$V_{\mathrm{cd}}=0.4\psi_{1}A_{\mathrm{cd}}f_{\mathrm{cu}}$$

As mentioned in Section 3.3, the steel fibers existing in the concrete dowel are able to enhance the shear capacity of the PBL connector through its bond with the concrete. Here, this effect is considered by introducing a reinforcement factor *K*_f1_ which is related to the volume content, length and diameter of fiber [38].

(8) $$K_{f1}=1+\psi_{2}V_{f}\frac{L_{f}}{\phi_{f}}$$

where *ψ*_2_ is the coefficient to be determined.

Finally, the contribution of the concrete dowels containing steel fibers to the shear capacity can be obtained as:

(9) $$V_{\mathrm{cd}}=0.4\psi_{1}\left(1+\psi_{2}V_{f}\frac{L_{f}}{\phi_{f}}\right)A_{\mathrm{cd}}f_{\mathrm{cu}}$$

#### 4.2.2. Contribution of Concrete End-Bearing

The concrete located at the end of perforated steel plates is able to provide a portion of the shear strength of the PBL connectors. The anti-shear contribution is determined by the contact area between the perforated steel plates and concrete, as well as the compressive strength of the concrete. Consequently, the shear resistance of the concrete end-bearing can be expressed as:

(10) $$V_{\mathrm{eb}}=A_{e}{}_{b}f_{\mathrm{cu}}$$

where *A*_eb_ denotes the contact area between the steel plate and concrete (mm^2^).

Similar to the calculation of the shear contribution of concrete dowels, considering the pressure of the surrounding concrete and the strengthening effect of the steel fibers on the strength of concrete end-bearing, here *V*_eb_ can be further expressed by adopting the strength enhancement factor as:

(11) $$V_{\mathrm{eb}}=\psi_{1}\left(1+\psi_{2}V_{f}\frac{L_{f}}{\phi_{f}}\right)A_{\mathrm{eb}}f_{\mathrm{cu}}$$

Consequently, the contribution of concrete dowels and concrete end-bearing can be combined as:

(12) $$V_{\mathrm{cd}}+V_{\mathrm{eb}}=\psi_{1}\left(1+\psi_{2}V_{f}\frac{L_{f}}{\phi_{f}}\right)\left(0.4A_{\mathrm{cd}}+A_{\mathrm{eb}}\right)f_{\mathrm{cu}}$$

#### 4.2.3. Contribution of Interface Bond

The perforated steel plates could contribute to the shear capacity of the PBL connector by means of the bond between it and the nearby concrete, and the contribution is related to the contact area and bond strength between the steel plates and concrete, which can be formulated as:

(13) $$V_{\mathrm{ib}}=A_{b}f_{b}$$

where *f*_b_ denotes the bond strength between the perforated steel plates and concrete (MPa), which is related to the square root of the concrete compressive strength as [15]:

(14) $$f_{b}=\psi_{3}\sqrt{f_{\mathrm{cu}}}$$

where *ψ*_3_ is the correlation coefficient to be determined.

Just as the enhancement influence of steel fibers on the shear capacity of concrete dowels, steel fibers also have an enhancement effect on the bond strength between the perforated steel plates and concrete, thus the enhancement factor *K*_f2_ is introduced like *K*_f1_ to reflect the effect as [38]:

(15) $$K_{f2}=1+\psi_{4}V_{f}\frac{L_{f}}{\phi_{f}}$$

where *ψ*_4_ is the coefficient to be determined.

In the end, the contribution of the interface bond to the shear capacity of the PBL connector is:

(16) $$V_{\mathrm{ib}}=\psi_{3}\left(1+\psi_{4}V_{f}\frac{L_{f}}{\phi_{f}}\right)A_{\mathrm{sp}}\sqrt{f_{\mathrm{cu}}}$$

#### 4.2.4. Contribution of Penetrating Rebar

The penetrating rebars are subjected to both bending moment and shear force in the working phase. According to existing studies, the shear resistance of the penetrating rebars is not affected by the concrete strength as well as the steel fibers, and is only related to the shear strength and cross-sectional area of the rebars [6]. Therefore, referring to the calculation of shear contribution of concrete dowels, the shear contribution of penetrating rebars could be calculated as:

(17) $$V_{\mathrm{pr}}=2A_{\mathrm{pr}}f_{s}$$

where *A*_pr_ denotes the cross-sectional area of penetrating rebar (mm^2^); and *f*_s_ denotes the shear strength of penetrating rebar (MPa), which can be expressed by *f*_s_ = *f*_y_/$\sqrt{3}$.

Furthermore, there would be shear deformation in the penetrating rebars after the force is applied, and the concrete in the deformation direction of the rebars can prevent their further deformation to enhance the shear capacity of the connectors. It can be speculated that the degree of enhancement to the shear capacity on the penetrating rebars is related to the deformation degree of rebars, hence it is reasonable to introduce a coefficient *ψ*_5_ to be determined to reflect this effect as [6]:

(18) $$V_{\mathrm{pr}}=1.15\psi_{5}A_{\mathrm{pr}}f_{y}$$

### 4.3. Establishment and Validation of Shear Capacity Equation

Ultimately, the calculation equation for the shear capacity of PBL connectors can be established as follows, where *ψ*_1_, *ψ*_2_, *ψ*_3_, *ψ*_4_ and *ψ*_5_ are the coefficients to be determined by experiment data.

(19) $$V_{u}=\psi_{1}\left(1+\psi_{2}V_{f}\frac{L_{f}}{\phi_{f}}\right)\left(0.4A_{\mathrm{cd}}+A_{\mathrm{eb}}\right)f_{\mathrm{cu}}+\psi_{3}\left(1+\psi_{4}V_{f}\frac{L_{f}}{\phi_{f}}\right)A_{\mathrm{sp}}\sqrt{f_{\mathrm{cu}}}+1.15\psi_{5}A_{\mathrm{pr}}f_{y}$$

After the regression analysis was performed, the values of the coefficients *ψ*_1_, *ψ*_2_, *ψ*_3_, *ψ*_4_ and *ψ*_5_ can be obtained as 1.205, 0.427, 0.016, 1.056 and 3.037, respectively. Accordingly, the shear capacity of the PBL connectors with SFHSC can be derived as:

(20) $$V_{u}=\left(1.205+0.515V_{f}\frac{L_{f}}{\phi_{f}}\right)\left(0.4A_{\mathrm{cd}}+A_{\mathrm{eb}}\right)f_{\mathrm{cu}}+\left(0.016+0.017V_{f}\frac{L_{f}}{\phi_{f}}\right)A_{\mathrm{sp}}\sqrt{f_{\mathrm{cu}}}+3.493A_{\mathrm{pr}}f_{y}$$

**Figure 10:**
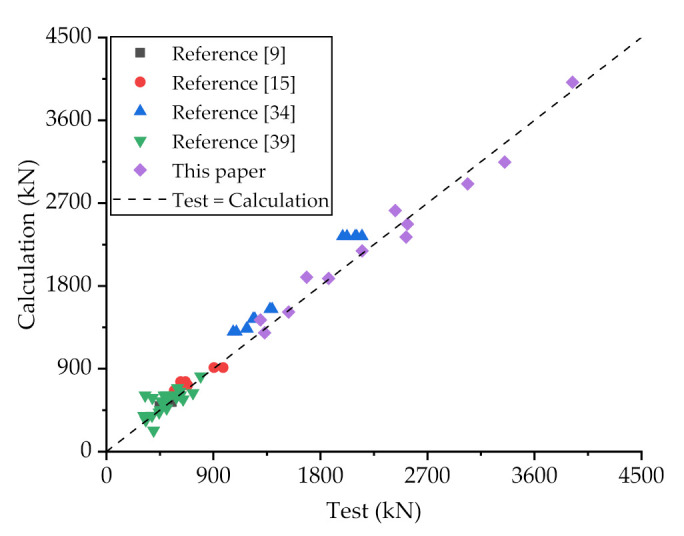
Comparison between test and calculation results of different literature.

The prediction results based on Equation (20) for the shear capacity of each specimen in this study are presented in Table 4, with the mean value of the ratio of calculation results to experimental data of 0.98. It can be seen that the results obtained from Equation (20) and the experimental results are in good agreement and Equation (20) can accurately estimate the shear capacity of PBL connectors with SFHSC. This is mainly due to that this formula fully and reasonably accounts for the anti-shear contribution of concrete dowels, concrete end-bearing, interface bond and penetrating rebars, as well as the enhancement effect of steel fibers to the shear capacity of PBL connectors. Furthermore, the prediction results based on Equation (20) for the experiments in other literature [9,15,34,39] are presented in Figure 10 (specimens in the literatures [9,34,39] adopting conventional concrete and those in the literature [15] adopting UHPC). It can be seen that the prediction results are quite close to the experimental data. Specifically, the mean value and the standard deviation of the ratio of calculation results to experimental data are 1.08 and 0.15, respectively, and the coefficient of determination and the Pearson coefficient are 0.90 and 0.93, respectively. This indicates that the equation established in this paper has outstanding applicability. Therefore, the formula proposed in this study could be utilized to predict the shear capacity of PBL connectors and provide guidance for the design of PBL connectors in steel-concrete composite structures in practical engineering.

## 5. Conclusions

This paper focuses on the study of the shear performance of PBL connectors applying SFHSC. With concrete strength, diameter of penetrating rebar and number of holes of PBL connectors being considered, 12 specimens were designed and tested, based on which the parameter influence mechanism was explored and the shear capacity equation for PBL connectors with full consideration of each shear contribution is established. The main conclusions drawn from this study are as follows:

(1) PBL connectors employing SFHSC primarily experience one or more of the failure modes of concrete dowel crushing, penetration reinforcement fracture and steel plate yielding, while essentially no severe cracking of the concrete slab occurs, which may appear in the PBL connectors employing conventional concrete. Moreover, as shear failure of the penetrating reinforcement occurs, it is often accompanied by failure of the concrete dowel. The full load carrying capacity of the member that does not fail is not fully utilized when the connector fails, and the desirable design is that the concrete dowel, the penetrating rebars, the steel plate and the concrete slab all achieve their full load carrying capacity.
(2) The shear capacity of PBL connectors increases with the increase in concrete strength, diameter of penetrating reinforcement and number of holes and the influence degree of the change in the number of holes is the most significant. In the design, the increase of these parameters is effective in increasing the shear capacity of the connectors. The continuous growth of these parameters cannot bring a constant and significant increase in the shear capacity of PBL connectors, and the mutual adaptation of the parameters of PBL connectors should be ensured in the design process. Additionally, the random distribution of steel fibers in concrete can effectively inhibit the expansion of old cracks and the formation of new cracks, and then enhance the ductility and shear capacity of the specimens.
(3) One general prediction formula for the shear capacity of PBL connectors was established based on regression analysis of test data. The proposed formula accounts for the contribution of concrete dowels, concrete end-bearing, interface bond between perforated steel plates and concrete and penetrating rebars to the shear capacity of the PBL connectors, as well as the reinforcing effect of the steel fibers on the material properties. The comparison of the prediction results obtained using the proposed formula with the experimental results demonstrates the excellent accuracy and applicability of the proposed formula, which can be used for the prediction of the shear capacity and provide a reference for the design of PBL connectors.

## Figures and Tables

**Table 1:** Test specimen details.

Specimen Label	Concrete			*d* (mm)	Perforated Steel Plate (mm)
	Nominal Strength (MPa)	*f*_cu_ (MPa)	*E*_c_ (GPa)		
C50-D12-N2	50	52.3	37.5	12	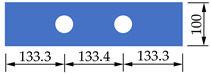
C50-D16-N2	50	50.5	37.1	16	
C80-D12-N1	80	83.3	43.2	12	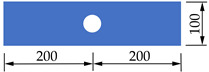
C80-D16-N1	80	82.7	42.8	16	
C80-D12-N2	80	81.2	43.7	12	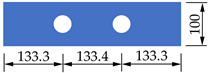
C80-D16-N2	80	80.3	42.5	16	
C80-D20-N2	80	80.9	42.8	20	
C80-D12-N3	80	79.8	42.1	12	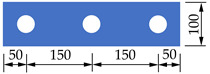
C80-D16-N3	80	81.3	42.8	16	
C80-D20-N3	80	81.4	43.2	20	
C100-D12-N2	100	103.7	46.0	12	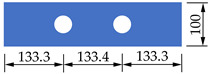
C100-D16-N2	100	104.2	46.2	16	

Note: C denotes the concrete strength; D denotes the diameter of penetrating rebar; N denotes the number of holes on perforated steel plate. For example, label C80-D12-N2 indicates 2 holes for each steel plate, 12 mm diameter for the reinforcement bars and a nominal compressive strength of 80 MPa for the surrounding concrete.

**Table 2:** Test results of all specimens.

Specimen Label	*P*_max_ (kN)	*δ*_max_ (mm)	*P*_rk_ (kN)	*δ*_rk_ (mm)	*K*_s_ (kN·mm^−1^)
C50-D12-N2	1295.3	5.63	1165.8	23.42	3847.4
C50-D16-N2	1684.3	4.74	1515.9	19.33	4972.2
C80-D12-N1	1330.3	6.23	1197.3	15.65	3818.6
C80-D16-N1	1532.5	18.93	1379.3	22.86	3951.2
C80-D12-N2	1868.9	10.42	1682.0	16.15	6511.5
C80-D16-N2	2520.2	8.21	2268.2	19.49	6131.6
C80-D20-N2	3038.7	9.08	2734.8	24.34	6183.5
C80-D12-N3	2532.2	6.99	2279.0	16.80	7468.5
C80-D16-N3	3350.2	9.81	3015.2	18.83	8531.2
C80-D20-N3	3920.7	8.65	3528.6	18.73	9529.0
C100-D12-N2	2150.9	10.71	1935.8	18.35	8501.2
C100-D16-N2	2429.6	7.97	2186.6	25.98	6050.9

**Table 3:** Typical prediction formulas for the shear capacity of PBL.

Authors	Prediction Equation		Notation
Oguejiofor and Hosain [8]	$V_{u}=4.5h_{sc}t_{sc}f_{ck}+0.91A_{tr}f_{y}+3.31nD^{2}\sqrt{f_{ck}}$	(1)	*V*_u_: shear capacity of connector (N) *h*_sc_: height of steel plate (mm) *t*_sc_: thickness of steel plate (mm) *f*_ck_: concrete prismatic strength (MPa) *f*_cu_: concrete cubic strength (MPa) *f*_y_: yield strength of rebar (MPa) *A*_tr_: area of rebar (mm^2^) *A*_b_: contact area between steel plate and concrete (mm^2^) *n*: number of holes *D*: diameter of hole (mm) *τ*_b_: residual bond stress (MPa) *V*_f_: volume content of steel fibers *L*_f_: length of steel fibers (mm) *φ*_f_: diameter of steel fibers (mm)
Ahn et al. [34]	$V_{u}=2.76h_{\mathrm{sc}}t_{\mathrm{sc}}f_{\mathrm{ck}}+1.06A_{\mathrm{tr}}f_{y}+3.32n\pi {\left(\frac{D}{2}\right)}^{2}\sqrt{f_{\mathrm{ck}}}$	(2)	
JTGD64-2015 [35]	$V_{u}=1.4\left(D^{2}-d^{2}\right)f_{\mathrm{cu}}+1.2d^{2}f_{\mathrm{sd}}$	(3)	
He et al. [15]	$V_{u}=\tau_{b}A_{b}+(1.06+0.07V_{f}\frac{L_{f}}{\phi_{f}})\frac{\pi \left(D^{2}-d^{2}\right)}{4}f_{\mathrm{cu}}+2.09A_{\mathrm{tr}}f_{y}$	(4)	

**Table 4:** Comparison between test and calculation results.

Specimen Label	*V*_u_ (kN)	Equation (1)		Equation (2)		Equation (3)		Equation (4)		Equation (20)	
		*V*_cu_ (kN)	*V*_cu_/*V*_u_	*V*_cu_ (kN)	*V*_cu_/*V*_u_	*V*_cu_ (kN)	*V*_cu_/*V*_u_	*V*_cu_ (kN)	*V*_cu_/*V*_u_	*V*_cu_ (kN)	*V*_cu_/*V*_u_
C50-D12-N2	1295.3	911.9	0.70	741.0	0.57	1080.6	0.83	652.4	0.50	1127.4	0.87
C50-D16-N2	1684.3	1168.1	0.69	1039.4	0.62	1470.0	0.87	1236.0	0.73	1602.0	0.95
C80-D12-N1	1330.3	1068.8	0.80	763.4	0.57	686.0	0.52	483.6	0.36	1291.8	0.97
C80-D16-N1	1532.5	1196.9	0.78	912.6	0.60	869.5	0.57	769.7	0.50	1516.5	0.99
C80-D12-N2	1868.9	1233.7	0.66	955.4	0.51	1372.0	0.73	861.8	0.46	1882.1	1.01
C80-D16-N2	2520.2	1489.8	0.59	1253.7	0.50	1739.0	0.69	1442.0	0.57	2331.4	0.93
C80-D20-N2	3038.7	1819.2	0.60	1637.4	0.54	2211.0	0.73	2188.0	0.72	2909.2	0.96
C80-D12-N3	2532.2	1398.5	0.55	1147.3	0.45	2057.9	0.81	1240.0	0.49	2472.4	0.98
C80-D16-N3	3350.2	1782.7	0.53	1594.8	0.48	2608.6	0.78	2114.4	0.63	3146.4	0.94
C80-D20-N3	3920.7	2276.7	0.58	2170.3	0.55	3316.5	0.85	3238.5	0.83	4013.0	1.02
C100-D12-N2	2150.9	1459.7	0.68	1098.3	0.51	1576.7	0.73	888.2	0.41	2181.2	1.01
C100-D16-N2	2429.6	1715.9	0.71	1396.6	0.57	1928.0	0.79	1466.3	0.60	2620.0	1.08
Average	—	—	0.66	—	0.54	—	0.74	—	0.57	—	0.98

## Data Availability

Data is contained within the article.
